# The Out-Door Treatment of Phthisis at Cromer

**Published:** 1898-04-02

**Authors:** 


### THE OUT-DOOR TREATMENT OF PHTHISIS AT CROMER.

Cromer has the reputation of being a cold place, and many sarcastic writers have poked fun at it on that account. But it is of interest to observe that Dr. Burton-Fanning, in summing up his account of the open-air treatment of phthisis at the convalescent home there, says that the temperature charts show distinctly that more improvement was effected in the cooler months than in the summer. "It was quite remarkable to notice in 1896 how all the cases then under treatment simultaneously manifested decline of fever when the hot month of August was passed." One of the first results of the out-door life was generally improved appetite. This was fairly constantly noticed within a few days. Night sweats were soon diminished, often before there was any drop in the evening temperature. In all the febrile cases except one the adoption of the open-air life was followed by decline of the fever. The time taken to produce defervescence varied greatly, and it would seem that at least a month may elapse before the slightest improvement is seen even in cases that eventually do well. Febrile patients whose temperatures do not fall below about 99·6 deg. F. in the mornings are held [not to be suitable for strict open-air treatment, the disease being probably associated with some inflammatory affection, such as pneumonia, pleurisy, or bronchitis. Until morning temperature falls they should be confined to bed in a well-ventilated room, but afterwards they do exceedingly well under the strict open-air *régime.*

